# Discretion in the Performance of Dental Operations

**Published:** 1892-05

**Authors:** F. W. Sage

**Affiliations:** Cincinnati, O.


// THE DENTAL REGISTER.

VOL. XLVI.] MAY, 1892. [No. 5.

### COMMUNICATIONS.

### Discretion in the Performance of Dental Operations.

BY F. W. SAGE, D.D S., CINCINNATI, O.

Read before the Mississippi Valley Dental Society March 10th, 1892.

A professor in a certain dental college once said before his class that a man is rather likely to discover after ten years of practice that he knows nothing about dentistry. This statement if accepted literally might well discourage the beginner. We take it however to signify that the accumulated experiences of ten years of practice bring a man to a point of view from which he very likely discovers that he has not always performed operations intelligently; that is, with regard for or knowledge of their probable outcome.

It is a singular fact that notwithstanding the experiences of those who have preceded us, in any walk of life, may have been precisely the experiences which we come later to recognize as our own, most of us need to stumble and flounder and find out for ourselves before we arrive at the point of deriving practical benefit from the suggestions of those who tried to warn us. So much unconsciously escapes the memory of even the most devoted reader of standard text-books. So much which on the printed page appears plain and simple enough, becomes complicated and perplexing when it confronts the student later in his every day practice, that the wonder is that any but the most talented achieve even partial success. The tyro is from the very first skeptical as regards the statement that theory and practice are at all widely separated from each other. He cannot comprehend how it should be probable or possible to stumble in the performance, after a clear elucidation of the principles upon which any operation or course of treatment is to be performed or carried out. He is not even aware of the difficulties which have beset his instructors in determining which text-books are best adapted to the purpose of fully enlightening him. He is not conscious how futile would be his own unaided efforts to select among various text-books the particular one best calculated to yield him the information he needs. It is only after years of practice whereby he becomes practically familiar with the subjects of which they treat, that he acquires that facility in criticising which enables him on turning over those books to discover that a text-book clear, explicit, and thorough as to necessary details, is not always to be found in his collection. Here he discovers something implied, there something to be supplied, both of which quite escaped his notice in his earlier reading. He went out from the dental college, as he supposed, thoroughly equipped for practice, and now after ten years of practice he returns to his text-books to discover a thousand things written between the lines of which he had never dreamed.

But after all, give a young man of studious habits, quick apprehension, and added to these, as by no means least in importance, a retentive memory, and it ought not to be recorded of him after he has had ten years of practice that he knows nothing of dentistry. His experience ought, during that time, to have been valuable to him, chiefly as illustrations to impress still more firmly what he had learned from his reading, not as instruments for the correction of mistaken apprehensions. But precisely here the difficulty arises, to-wit: that in the beginning the student does not know, nor does his instructor know infallibly, what it is that he most needs to know. Still more obvious is it that many fail to seize upon and improve by the teaching of the passing experience, so that being forewarned they may be forearmed against a repetition of an error. Instead of sitting down to patiently consider the cause and source of their error, they pass it by as something not likely soon to recur in practice, promising later when leisure affords the opportunity to give it proper attention. To men of this temperament the convenient time never comes, and they fall into the train of the non-progressive.

Discretion in the performance of operations in dental practice may be an inherent trait of character in the less skilled dentist, whereby he becomes practically equal in ability to another of finer manipulative attainments, but of uncultured or hasty judgment. Defects of judgment are more likely to characterize the man of an imitative mind than one of an inventive turn, for the inventive mind is constantly inquiring as to the why and wherefore, whereas the imitative mind turning instinctively to familiar methods, is constrained to make the best of those methods, even though a glimmering suspicion of their inadequacy awakens distrust. No other resources present. But a broader view of means possible of acquirement opens before the man of inventive resources. Conscious of his latent powers he boldly announces "I will find a more effective way," where the mere imitator shrinks from considering a probable failure, and takes refuge behind the plea that no other method than the one commonly approved is at his command. The disposition to ask of himself "Why may not I find a better way" marks in the dentist a degree of mental alertness which is a kind of guarantee of his avoiding error. It is, of course, not a positive check against error, because the principle upon which he projects his invention may, after all, fail. Still, the constitution of such a mind is sure to be such as to detect flaws, if they exist, in current methods, and in this alone is an important element of progress. If the man occasionally makes a mistake, the sum of all his errors is likely to be more than offset by the aggregate of instances in which he points out in advance the false premises upon which others project vaunted inventions of appliances or methods of practice. He is not usually one to endorse a method or appliance on the word of another, nor is he, on the other hand, one of the many who decry without sufficient reasons a half-tried appliance or method.

But while there is, and must be, differences in gifts whereby ends are to be attained, there need be inferiority on the part of no one of intelligence in clearly recognizing conditions calling for specified courses of procedure in given cases. The ability to pause and thoughtfully consider, to forecast the probable out come of an operation, to estimate the patients' ability to bear it, ought to appear in the case of any and all dentists of ten years' experience.

The extension of his influence in the community, his hold upon the confidence of his patients, the widening of the field of his practice, in short, the measure of his success in coming years depends not merely upon the dentist's manipulative skill, but far more upon the careful cultivation of his judgment as to what to do and what to leave undone, for in this age of fierce competition no man holds an undisputed field ; family is linked to family among his patrons and he who through heedlessness makes a serious error may never learn the full extent of its adverse influence.

We hold then that the first requisite to the cultivation of proper habits of discretion in dental practice is a certain degree of incredulity as regards what one hears or reads. The more thoroughly honest at heart a man is the more particularly he needs to avoid the danger of accepting plausible statements from the platform and in the pages of dental journals. Not all of us are so constantly mindful as is the sagacious editor of the fallacies which are liable to characterize the writings of those who contribute to the journals, or we might be led as they are, to question their being duly accredited and responsible messengers of truth. Not that we bring any harsh accusations against the body of contributors, presumably as honest in their motives as we ourselves are : but access to the journals is easy, perhaps too easy, and some who write are too well satisfied with the achievement of having written at all to exercise a severely critical faculty in reviewing their finished work. The time is no doubt near at hand when all this will be changed, for with the accession of more liberally educated men to the ranks of the profession the number of able writers will be vastly multiplied ; the dental journal will no longer consent to serve skim-milk when an abundance of cream is to be had, and only carefully revised productions, sound in doctrine and calculated to edify will be accepted for publication. But at the present time some of our journals, aye, and some of our text books too, need to be read with a critical regard for the vanities and other petty foibles of authors who in the heat of composition forget accuracy, explicitness and fidelity to recognized principles.

Well do we need to bear in mind the legend, "Observe, compare, reflect, record."

There are many enthusiasts in the dental profession, men who though not exactly "swept to and fro by every wind of doctrine," are still not sufficiently self-contained, not sufficiently well poised to escape the infection of half-matured ideas and suggestions coming from unsanctioned authorities. The danger has been clearly recognized and provided against by an undertaking to sift out the kernels of wheat from the chaff and republish what is really valuable in the form of a compendium. While this is probably not intended as an affront to the intelligence of the mass of the profession, many there are who will elect to do their own winnowing, either through distrusting the ability of any one to select for them, or through a feeling that there is a certain advantage to be derived from considering the obvious errors of those whose contributions would be certain of being rejected from the compendium. Nor is this last consideration one to be lightly set aside, for a view of error is essential to stimulate progress, to provoke inquiry, to suggest improvement. Still the fact remains that he who compiles a compendium does so in the capacity of an editor and fulfills an important office in bringing to the attention of some who will not read widely what is passing in the world of dental letters. The product of his labor still remains amenable to the injunction to "observe, compare, reflect, record."

From this general view of our subject we come now to a practical consideration of the things which either contribute to the dentist's success in his profession or lead the way to his failure. Discretion in the performance of dental operations implies an intimate knowledge of the patient's health, temperament, daily habits of life, and many other things which come to the attention of the thoughtful observer. We premise that the dentist building up a practice needs to remember that one failure advertises him unfavorably in undue proportion to a dozen successful operations. He needs constantly to bear in mind that his patronage is not to be solely from those who come and go and never return, but from those who are almost sure to return with fillings lost, plates broken, and new decays. He needs to be impressed with the importance of foreseeing, in the course of events, what will probably happen to this filling or that plate, or the other piece of bridge work, and to fail on the side of predicting possible failure rather than yielding to a sanguine impulse to promise permanent success. It requires a degree of moral courage, which perhaps few possess, boldly to announce in advance of what promises to be a paying operation, that it will probably fail utterly, in three, five or ten years. Such a course may drive the applicant away from the office. And yet how often are patients dismissed after numerous sittings and trying operations, laboring under a delusive notion—which the operator dares not share—that they are done with dental operations for all time? Better far to have told them in the first place what you suspected. Still more unfortunate the case of the young dentist sanguine of his ability to accomplish the miracle, when he discovers years afterwards, his failure.

Discretion in the performance of dental operations appertains to a choice of time and occasion for performing them. The patient comes in announcing that he or she is not feeling well; must be back to the dressmakers or the counting-room in forty minutes; or the dentist himself is not feeling well, or has only forty minutes to spare for a two hours, operation. Just at this stage the operator with real discernment and decision of character scores a success by declining to operate at the time. He perhaps disappoints his patient, but he is more than likely to find that he has gained increased confidence. The foregoing relates perhaps more distinctly to management of the dentist's patients. Now for some of the instances of failure. A large iron-jawed man comes in complaining that his fillings are wearing out. He shows the six anterior teeth, upper and lower—twelve in all—bushed with gold. At least he reminds you that you bushed them three years before. The teeth proper project about a line above the gum borders. All of the gold in the lower six, excepting that in the retaining grooves, is gone. The upper six are battered all out of shape. You find by referring to your ledger that you finished those fillings with gold and platinum folds, and you recall that you told him that they ought to last twelve or fifteen years. He recalls that he paid you $200 for the fillings. There they are, a brilliant array of ruins. You ask him: "where are the plates I inserted to supply the loss of the back teeth?" "Oh I never wore them after the first day." "Didn't I caution you that you must wear them, or the fillings in front would be likely to give way?" "No, I don't think you did." Nor did you, because you had in reality, no such misgivings. You thought that the gold and platinum cappings would stand any abuse. But what most chagrins you is the discovery that most of the teeth are loose; pus oozes out around them. The man had catarrh when you performed the operation, and you noticed the fact, and in the abscence of any certain knowledge that catarrh may involve the teeth, you went ahead without particularly considering this possible, even probable outcome. Now you wish that you had extracted those teeth and made the man a full denture. You know that more partial sets of this description are worn in wash-stand drawers than in people's mouths, and yet you made those partial sets, hoping that this case would prove an exception.

Case No. 2: A patient comes in for whom you filled half a dozen proximal cavities in incisors, a year before. Molars and bicuspids in both jaws are missing. Fillings out, much to your astonishment. The teeth are widely separated and incline inward. You fill them again, taking extraordinary pains. In another year the patient returns; filling out the second time. And so it goes on, until finally you wake up to a realization that they have been *ground* out. The teeth have been doing double work, as grinders and incisors proper. The cavities being difficult of access you were unable to mallet the fillings throughout, hand pressure was largely relied on; a filling lacking in the quality of density was the result, and constant attrition caused their disintegration. You recall the fact that you advised wholesale extraction and a full denture, in the first place; but the patient demurred, and you weakly yielded instead of insisting on being the "doctor."

Case No. 3:—An Irish cook. You will know better next time.

Case No. 4:—Old lady of seventy. Has been edentulous for twenty years. Children insist on her having a set of teeth. No perceptible alveolar ridge below. Lips deeply retracted. You make full dentures with ample restoration, by means of "plumpers" of lost tissue. Patient delighted. Wears teeth for six months in little blue china closet. Too much of a mouthful. You make another set, using similar teeth and no "plumpers." Old lady waylays you at church "mite" societies, county fair, funerals, etc. Wants her money back. Moral: Don't undertake to insert teeth for old people.

---

DISCUSSION ON DR. SAGE'S PAPER.

*Dr. H. A. Smith.*—I regret I was not here when the paper was read. Dr. Sage, I think, refers to his student life, but not to the student life of the present day. It used to be the theory of our friend, the late Dr. Berry, that nobody ought to practice dentistry till he had been in a dental office ten years and had been thoroughly trained and instructed in the direction of discrimination in the performance of dental operations. It does not require ten years to acquire some discrimination in the performance of our operations. There is no class of professional men who come up so well prepared as the graduates of our dental colleges at the present day. About text books: there is a great lack of text books; the student has no text books that are up to date. When shall we have good text books? When there is a demand for them. Then, at present this knowledge must come from observation or from the teacher. The best thing we have at the present day in way of books are our dental journals. We have a class of writers that are giving us excellent articles.

Dr. Sage goes into the question of inheritance, those who are natural born dentists. They are the most dangerous class. Dr. Sage goes on to speak of failures in practice where teeth are filled on the cutting edge to be broken down in a few years. Why do they fail? The impact in use disintegrates from the enamel, or the enamel gives way under the force brought upon it and the filling comes out. That is no lack of discrimination in the making of these fillings and the dentist should not be expected to do them over again without remuneration. The last part of the paper refers to an old lady. There is no lack of discrimination made in making teeth for old ladies, the difficulty is in getting them to be persistent in learning to wear them. A gentleman said to me some time ago: "I do not want these teeth, but my physician says I must have them and my family wants me to have them." I made the teeth and he took them home and put them away in the bureau-drawer but would not try to wear them. Our young practitioners do not need ten years now to learn how to discriminate; they do discriminate and this is on account of their thorough college training.

*Dr. Sage.*—I took the ground, or assumed, in my paper that a man ought to know a good deal about dentistry after being in practice ten years, if not it is his own fault; but some go along without making notes of their own failures, and consequently they learn nothing as they proceed. As regards the responsibility of the dentist our patients expect more from us than we are willing to give. Warn them not to expect too much from their dentists, yet it is not an easy matter to make them understand that we are not responsible for the fillings. We are held more responsible than the physician.

When the physician gives a prescription he tells the patient if it does not help or give relief to come back again. I maintain, therefore, that the dentist ought not to promise too much, so that people can come back in ten years expecting to have their fillings replaced without charge. It is better to discourage a patient on partial dentures. As to the wearing away of a gold filling tipped with platinum, I was never cautioned before I used it. In the case of the gentleman who came in with all the teeth gone except five or six and wanted them filled, I wish I had pulled out the teeth and put in a full set.

